# Blood–brain barrier and gut barrier dysfunction in chronic kidney disease with a focus on circulating biomarkers and tight junction proteins

**DOI:** 10.1038/s41598-022-08387-7

**Published:** 2022-03-15

**Authors:** Leah Hernandez, Liam J. Ward, Samsul Arefin, Thomas Ebert, Agne Laucyte-Cibulskiene, GOING-FWD Collaborators, Louise Pilote, Colleen M. Norris, Valeria Raparelli, Alexandra Kautzky-Willer, Maria Trinidad Herrero, Karolina Kublickiene, Olof Heimbürger, Peter Barany, Lars Wennberg, Peter Stenvinkel, Karolina Kublickiene

**Affiliations:** 1 Department of Clinical Science, Intervention and Technology (CLINTEC), Division of Renal Medicine, Karolinska Institutet, 14186 Stockholm, Sweden; 2 Department of Clinical Sciences, Skåne University Hospital, Lund University, Malmö, Sweden; 3 Department of Transplantation Surgery, Karolinska University Hospital, Stockholm, Sweden; 4 McGill University Health Center and McGill University, Montreal, Canada; 5 University of Alberta, Edmonton, Canada; 6 University of Ferrara, Ferrara, Italy; 7 Medical University of Vienna, Vienna, Austria; 8 Universidad de Murcia, Murcia, Spain

**Keywords:** Biomarkers, Kidney diseases, Tight junctions

## Abstract

Kidney failure and associated uraemia have implications for the cardiovascular system, brain, and blood–brain barrier (BBB). We aim to examine BBB disruption, by assessing brain-derived neurotropic factor (BDNF), neuron-specific enolase (NSE) levels, and gut-blood barrier (GBB) disruption by trimethylamine N-oxide (TMAO), in chronic kidney disease (CKD) patients. Additionally, endothelial tight-junction protein expressions and modulation via TMAO were assessed. Serum from chronic kidney disease (CKD) female and male haemodialysis (HD) patients, and controls, were used to measure BDNF and NSE by enzyme-linked immunosorbent assays, and TMAO by mass spectrometry. Immunofluorescent staining of subcutaneous fat biopsies from kidney transplant recipients, and controls, were used to measure microvascular expression of tight-junction proteins (claudin-5, occludin, JAM-1), and control microvasculature for TMAO effects. HD patients *versus* controls, had significantly lower and higher serum levels of BDNF and NSE, respectively. In CKD biopsies *versus* controls, reduced expression of claudin-5, occludin, and JAM-1 were observed. Incubation with TMAO significantly decreased expression of all tight-junction proteins in the microvasculature. Uraemia affects BBB and GBB resulting in altered levels of circulating NSE, BDNF and TMAO, respectively, and it also reduces expression of tight-junction proteins that confer BBB maintenance. TMAO serves as a potential candidate to alter BBB integrity in CKD.

## Introduction

Chronic kidney disease (CKD) is a progressive irreversible systemic disease that affects around 10–15% of population worldwide^1^. As CKD progresses to kidney failure, suitable patients are prepared for renal replacement therapy (RRT), either kidney transplantation or dialysis. Uraemia has repercussions for other organs^2^, and is an established risk factor for premature cardiovascular diseases (CVD)^3,4^. CKD also serves as a model of accelerated ageing, leading to reduced physical functioning, increased frailty, vascular dysfunction, vascular calcification, arterial stiffness, increased systemic inflammation, and oxidative stress^5–7^. Additionally, CKD patients have a high prevalence of small vessel disease^8^, with microvascular damage contributing to cognitive changes related to dementia^9^. Indeed, the increasing prevalence of kidney dysfunction and neurological disorders are associated with higher morbidity and mortality, resulting in lower quality of life and higher cost of healthcare^10,11^. There is currently no treatment to ameliorate the CKD-related cognitive impairment, which is only partially reversible after kidney transplantation^12^. Thus, further investigations are required to elucidate the reason why the uraemic milieu promotes central nervous system manifestations, such as cognitive impairment and depression.

The central nervous system (CNS) is protected by the blood–brain barrier (BBB). The BBB is comprised of endothelial cells, pericytes, astrocyte foot processes, and basement membrane that protects and regulates the CNS environment by restricting permeability and ensuring the selective exchange of molecules between blood and the CNS^13^. The impairment in BBB function may further lead to increased vascular permeability allowing entry of toxic substances into the CNS, resulting in damage that may manifest as depression and cognitive impairments that are similarly observed in the cognitive dysfunction in patients with Alzheimer’s disease^14^. There is limited available data on the prevalence of Alzheimer’s disease in CKD patients, but it has been reported that vascular dementia is more likely the cause of cognitive dysfunction rather than Alzheimer’s disease in kidney disease^15^.

Several studies suggested that impaired BBB function could be assessed by peripheral circulation analyses of neuron-specific proteins that may differ between the diseased and healthy conditions^16^, although little is known regarding sex specific differences. A number of candidate biomarkers for increased BBB permeability have been introduced, such as: brain-derived neurotrophic factor (BDNF) and neuron-specific enolase (NSE). BDNF regulates survival, growth, and maintenance of neurons^17,18^. Detected in systemic circulation, patients with reduced plasma BDNF concentrations had increased risk for developing CKD^17^, and lower BDNF levels were likewise correlated with higher depression scores^19^. NSE has been introduced as a promising biomarker of future brain-related vascular events and subclinical brain damage among asymptomatic hypertensive patients^20^.

Due to the difficulties in the acquisition of human brain tissues and subsequent isolation of brain microvessels, there has been limited research access to human brain endothelial cells. Thus, studies have aimed at modelling the BBB, although the inherent complexity of the BBB has limited the development^21^. Small-vessel disease in the brain is mirrored by small-vessel pathologies in other organs^22,23^. Thus, surrogate vessels may be useful in elucidating certain features of the BBB, such as tight junctions that are ubiquitously expressed between endothelial cells. Various tight-junction proteins have been identified, including, but not limited, to: claudin-5, occludin and junction adhesion molecule-1 (JAM-1). Claudin-5 is the dominant claudin present in BBB endothelial cells^24^. The first transmembrane tight-junction protein identified, occludin, has been shown to be increased in the peripheral circulation of animal models of cerebral ischaemia^25^, while JAM-1, located at the epithelial and endothelial tight junctions, has been shown to be involved in regulation of endothelial cell migration^26^.

Recently, the importance of gut-blood barrier (GBB) has been introduced to play an important role in respect to uraemia induced CVD outcomes. GBB has a physical barrier comprised of a monolayer of enterocytes connected via tight-junctions, physically similar to the BBB with its endothelial cells and tight-junctions. Trimethylamine N-oxide (TMAO) is a gut-microbiota metabolite derived from precursors choline, betaine, and carnitine, which can permeate the GBB^27^. However, less is known how TMAO could affect the maintenance of BBB integrity. Most recently, TMAO and its precursor TMA have been shown to affect the integrity of human brain microvascular endothelial cell monolayers, an in vitro model of the BBB, altering the paracellular permeability^28^. Elevated levels of TMAO are associated with poor survival in CKD^29,30^. TMAO has been positively correlated with atherosclerosis and that higher levels of TMAO increases cardiovascular events and all cause mortality^31^. In addition, the relationship between brain ageing and TMAO has been shown to induce age-related cognitive dysfunction in senescent prone mouse strain^32^.

We hypothesised that CKD is associated with BBB dysfunction and lessened tight-junction integrity. We aimed therefore to investigate potential BBB dysfunction under uraemic conditions. BBB permeability was assessed by measurements of CNS biomarkers in serum. In addition, gut-derived marker TMAO representing the GBB was also assessed. We also aim to examine if there are sex-specific associations and effects of the uraemic environment by evaluating mortality, as well as decline in cognitive function through self-reported depression. In addition, tight-junction integrity was examined by analysing the expression of claudin-5, occludin and JAM-1 using microvessels of subcutaneous fat biopsies from kidney failure (KF) patients and non-CKD patients as surrogate model of BBB. Furthermore, we also assessed if TMAO exposure of microvessels could affect the expression of the tight junction proteins.

## Results

Clinical characteristics of the HD patients and controls are summarised in Table 1. The age of the participants was comparable in all groups, median age of HD patients was 66 while control group median age was 64. As controls were randomly selected from the same geographical recruitment area as the patients they were not devoid of comorbidities, such as CVD and diabetes mellitus (DM). However, HD patients had significantly higher frequencies of both CVD and DM comorbidities than the control group. HD patients showed lower cholesterol, albumin and haemoglobin levels, and greater creatinine, CRP and IL-6 compared to controls (Table 1). Significantly higher number of HD patients were on medication with angiotensin-converting enzyme inhibitors/angiotensin receptors blockers, beta-blockers, and statins compared to controls (Table 1).

**Table 1:** Clinical characteristics of patient groups at baseline in non-chronic kidney disease (CKD) controls, haemodialysis (HD) used for peripheral biomarker assay.

	HD (n = 219)	Controls (n = 80)	*p* value
Age, years	66 (51–74), n = 207	64 (55–70)	0.4130
Males, *n* (%)	115 (56), n = 206	56 (70)	0.0282
Body mass index, kg/m^2^	23.9 (21.1–27.2), n = 206	25.4 (23.3–29)	0.0036
Cardiovascular disease, *n* (%)	130 (63), n = 207	7 (9)	< 0.0001
Diabetes mellitus, *n* (%)	51 (25), n = 207	4 (5)	0.0002
**Biochemistry**			
Cholesterol, mmol/L	4.4 (3.7–5.1), n = 201	5 (4.3–5.7), n = 79	< 0.0001
Triglycerides, mmol/L	1.6 (1.1–2.3), n = 203	(0.8–1.7), n = 79	< 0.0001
HDL, mmol/L	1.3 (1.1–1.7), n = 210	1.4 (1.2–1.7)	0.0019
Albumin, g/L	35 (33–37), n = 207	39 (37–41)	< 0.0001
Creatinine, μmol/L	771 (628–914), n = 207	79 (69–92)	< 0.0001
Haemoglobin, g/L	119 (111 – 127), n = 207	144 (136–149)	< 0.0001
hsCRP, mg/L	5.6 (2.4–17.0), n = 202	1.3(0.7–3.4), n = 70	< 0.0001
IL-6, pg/mL	7.8 (4.9–14.9), n = 207	2.0 (1.0–4.0), n = 28	< 0.0001
Ferritin, ng/mL	418 (233–635), n = 213	137 (87–204), n = 75	< 0.0001
**Medications**			
ACEi/ARB, *n* (%)	69 (33), n = 207	11 (14)	0.0009
Beta-blockers, *n* (%)	103 (50), n = 207	14 (18), n = 79	< 0.0001
Statins, *n* (%)	68 (33), n = 207	10 (13), n = 77	0.0009

Data are presented as median and quartile range (Q1–Q3). Categorial data are presented as frequency (%). Continuous data analysed by non-parametric Mann–Whitney *U* test, and categorical data analysed by χ^2^. *p* value < 0.05.

*ACEi/ARB* angiotensin converting enzyme inhibitors/angiotensin-receptor blockers, *CKD* chronic kidney disease, *HD* haemodialysis, *HDL* high-density lipoprotein, *hsCRP* high-sensitivity C-reactive protein, *IL* interleukin.

### Peripheral biomarkers

**Figure 1:**
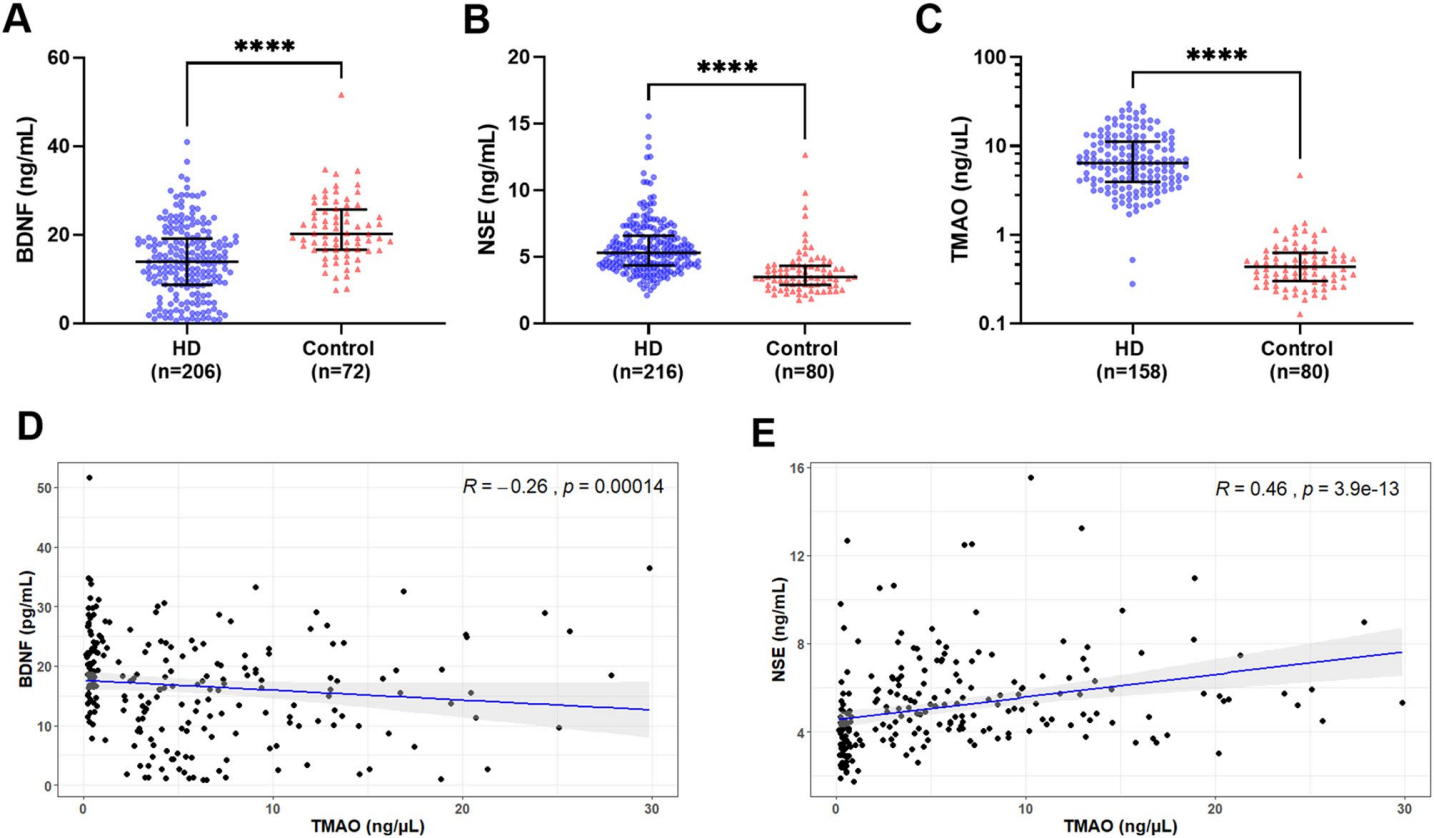
Analysis of serum biomarkers in haemodialysis (HD) and non-CKD controls (control). (**A**) Brain-derived neurotrophic factor (BDNF), (**B**) Neuron-specific enolase (NSE) were measured by enzyme-linked immunosorbent assay (ELISA). (**C**) Trimethylamine N-oxide (TMAO) was measured by liquid chromatography-mass spectrometry (LC–MS). The number of samples (n) that each analyte was detected in is noted for each group. Data presented at median and interquartile range. Statistical significance, ***p < 0.001, ****p < 0.0001. (**D**) Spearman’s correlation between BDNF and TMAO levels. (**E**) Spearman’s correlation between NSE and TMAO levels.

Results for the peripheral biomarkers showed BDNF levels were lower in HD patients (14.0 ng/mL, IQR 8.7–19.2) than controls (20.2 ng/mL, IQR 16.7–25.7) *p* < 0.0001 (Fig. 1A). Circulating NSE was higher levels in HD patients (5.3 ng/mL, IQR 4.4–6.6) than in controls (3.5 ng/mL, IQR 2.9–4.3) *p* < 0.0001 (Fig. 1B). TMAO analysis showed higher levels in HD group (6.4 ng/μL, IQR 4.0–11.2) vs. controls (0.4 ng/μL, IQR 0.3–0.6) *p* < 0.0001. (Fig. 1C). Correlation analyses between TMAO and the BBB markers BDNF and NSE showed a significant negative (rs = − 0.26, p < 0.001; Fig. 1D) and positive (rs = 0.46, p < 0.0001; Fig. 1E) correlation, respectively. S100B assay, although not statistically significant showed a pattern with lower circulating levels in HD (31.6 pg/mL, IQR 9.4–186*)* compared to controls (87.2 pg/mL, IQR 13.3–749). S100B was detected in measurable concentrations in only 21% (n = 44) of HD patients, and 26% (n = 21) of non-CKD control group (Supplement Fig. S1).

**Figure 2:**
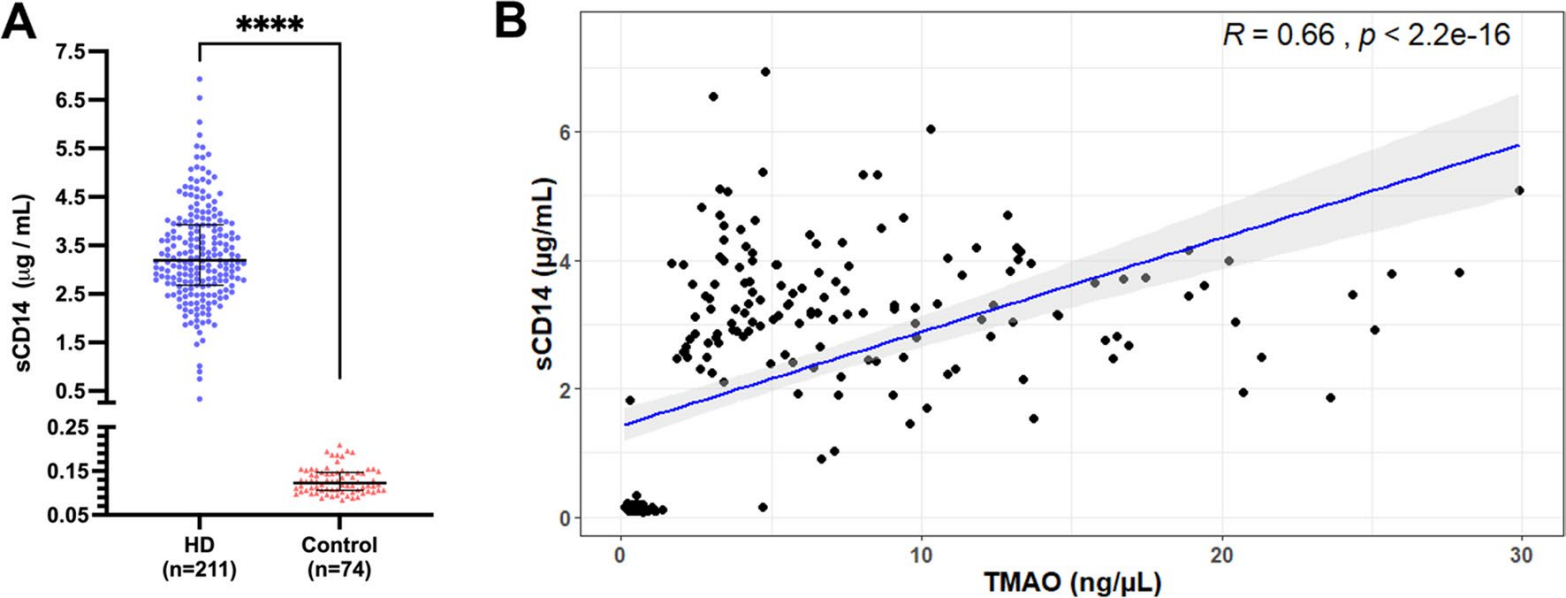
Analysis of soluble CD14 (sCD14) levels in haemodialysis (HD) and non-CKD controls (control). (**A**) Soluble CD14 levels. The number of samples (n) that each analyte was detected in is noted for each group. Data presented at median and interquartile range. Statistical significance, ***p < 0.001, ****p < 0.0001. (**B**) Spearman’s correlation between sCD14 and trimethylamine N-oxide (TMAO) levels.

To confirm GBB permeability soluble CD14 (sCD14) levels were assessed in serum samples from HD and non-CKD controls. For the current cohort of HD patients, sCD14 has been reported previously^33^, whereas for non-CKD controls sCD14 was measured herein. sCD14 results showed significantly greater levels in HD patients (3.18 μg/mL, IQR 2.68–3.90) than non-CKD controls (0.13 μg/mL, IQR 0.11–0.15) p < 0.0001 (Fig. 2A). Correlation analysis between TMAO and sCD14 showed a strong significant positive correlation (rs = 0.66, p < 0.0001; Fig. 2B).

### Sex differences in peripheral biomarkers

Analysis of the sex-specific abundances of peripheral biomarkers in each group revealed significant difference in TMAO levels only in controls (males 0.33 ng/μL, IQR 0.26–0.51; females 0.48 ng/μL, IQR 0.35–0.64; p = 0.036) (Supplementary Table S1). Biomarker levels for BDNF, NSE and S100B showed no difference between males and females (Supplementary Table S1). Sex disaggregated analysis for the tight junction protein expression was not possible due to the small sample numbers for biopsy donation.

### Correlation analyses

Correlation of the biomarkers NSE, BDNF, and TMAO with biochemical parameters for HD showed that serum levels of BDNF was negatively correlated with age (rs = − 0.17; p = 0.018) and serum ferritin (rs = − 0.27; p < 0.001), while NSE was positively correlated with inflammatory markers TNF (rs = 0.18; p = 0.044) and BNP (rs = 0.17; p = 0.020). TMAO was negatively correlated with age (rs = − 0.16; p = 0.022), and inflammatory marker IL-6 (rs = − 0.24; p = 0.003).

### Self-reported depression

**Figure 3:**
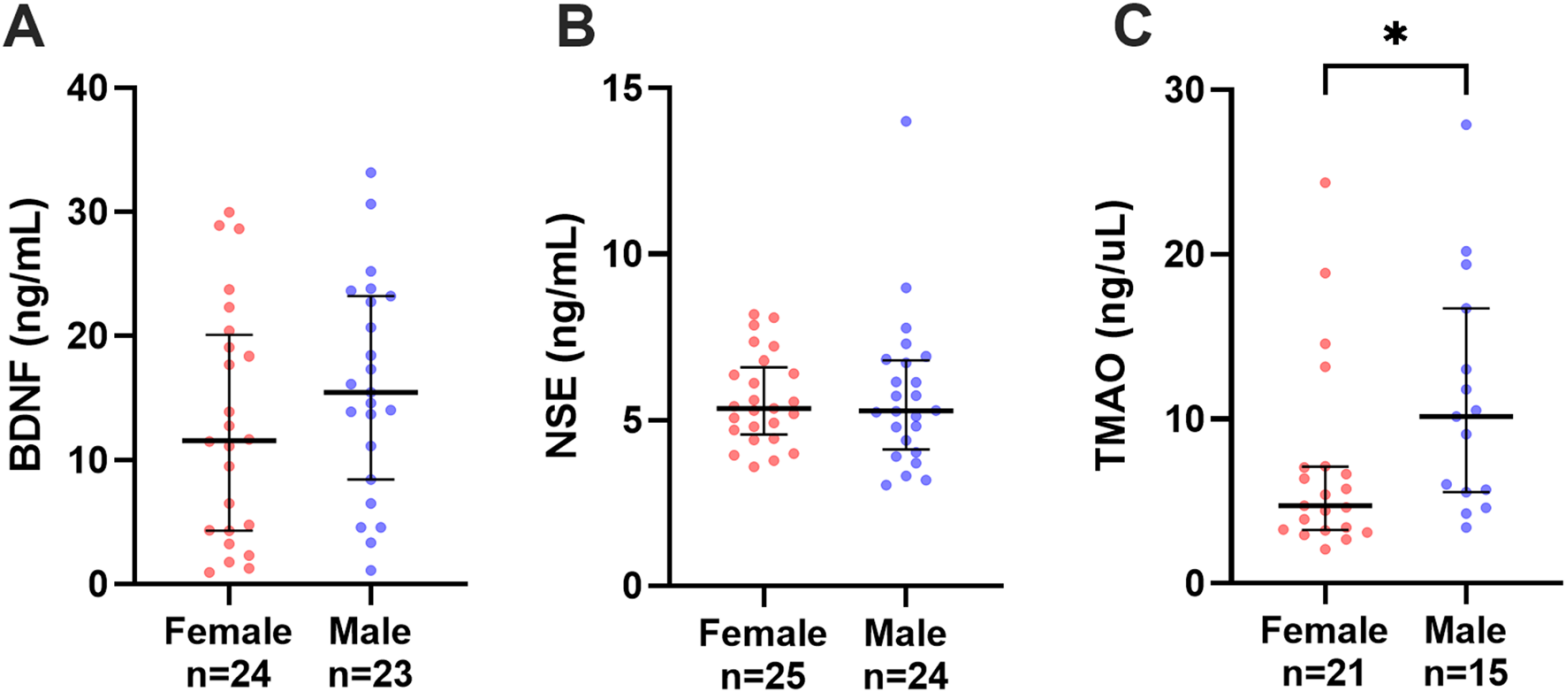
Sex-related differences in haemodialysis patients with self-assessed depression and serum concentrations of biomarkers; (**A**) brain-derived neurotrophic factor (BDNF), (**B**) neuron-specific enolase (NSE), (**C**) trimethylamine N-oxide (TMAO). The number of samples (n) that each analyte was detected in is noted for each group. Data presented at median and interquartile range when appropriate. Statistical significance, *p < 0.05.

There was no significant difference in the frequency of self-reported depression between females (n = 23) and males (n = 21, p = 0.247). However, for those taking anti-depressant medication there was a significant difference between females (n = 9/23, 39%) and males (n = 1/21, 5%, p = 0.003). No significant differences found in the levels of the biomarkers among those with or without self-reported depression in the HD group (Supplementary Fig. S2). In patients with self-reported depression, sex-related differences in biomarker levels among HD patients were observed showed significant results for TMAO only (Fig. 3A–C). In the HD group, lower TMAO levels were observed in females with self-reported depression vs. males (p 0.030) (Fig. 3C).

In HD patients with self-reported depression (n = 51), the biochemical parameters showed BDNF was negatively correlated with inflammatory marker ferritin (rs = − 0.44; p = 0.008). TMAO was positively correlated with BMI (rs = 0.35; p = 0.032).

### Mortality

**Figure 4:**
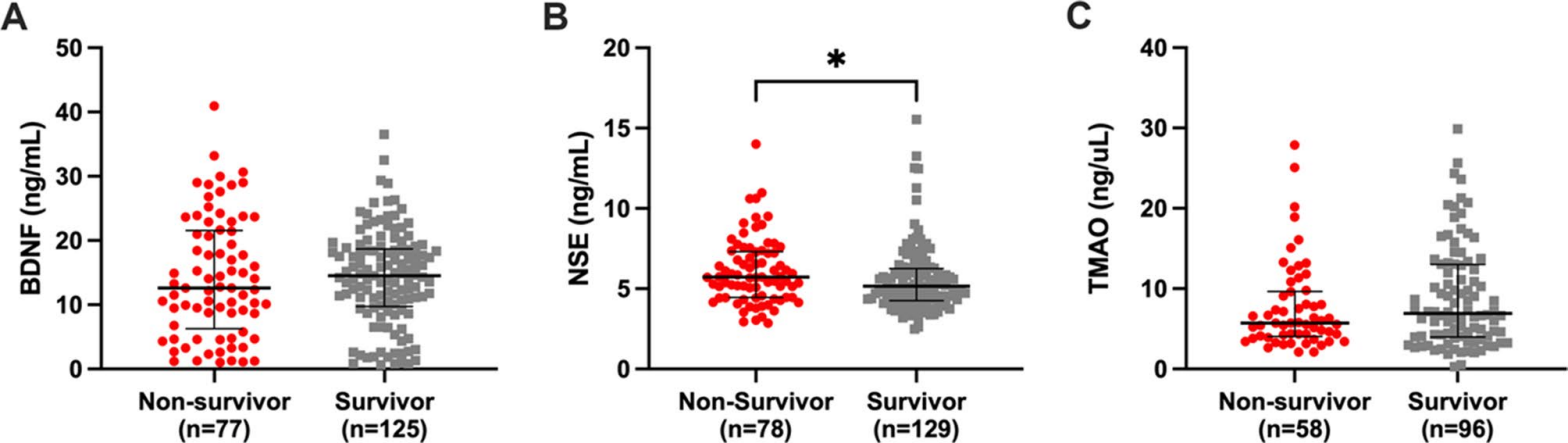
Mortality and serum concentration of biomarker in haemodialysis patients; (**A**) brain-derived neurotrophic factor (BDNF), (**B**) neuron-specific enolase (NSE), and (**C**) trimethylamine N-oxide (TMAO). The number of samples (n) that each analyte was detected in is noted for each group. Data presented at median and interquartile range when appropriate. Statistical significance, *p < 0.05, **p < 0.01.

At the conclusion of the five year follow-up period for HD patients, there were 139 patients (60.7%) alive and 85 (37.1%) patients that had died. Biomarker levels were compared between survivors vs non-survivors (Fig. 4A–C), where significantly higher levels of NSE in HD non-survivors (5.7 ng/ml) were observed compared to those who survived (5.2 ng/ml) (Fig. 4B). No sex-related differences were observed in the biomarker levels of BDNF, NSE and TMAO for survivors vs non-survivor among dialysis patients (Supplementary Fig. S3).

Survival analyses, using Cox regression models, for HD patients showed no independent association between mortality and NSE levels (HR 1.09; 95% CI 0.99–1.19; p = 0.069), BDNF levels (HR 1.00; 95% CI 0.97–1.04; p = 0.663), nor TMAO levels (HR 0.99; 95% CI 0.94 1.04; p = 0.712), with adjustments for sex, age, DM, and CVD.

### Tight-junction staining

**Figure 5:**
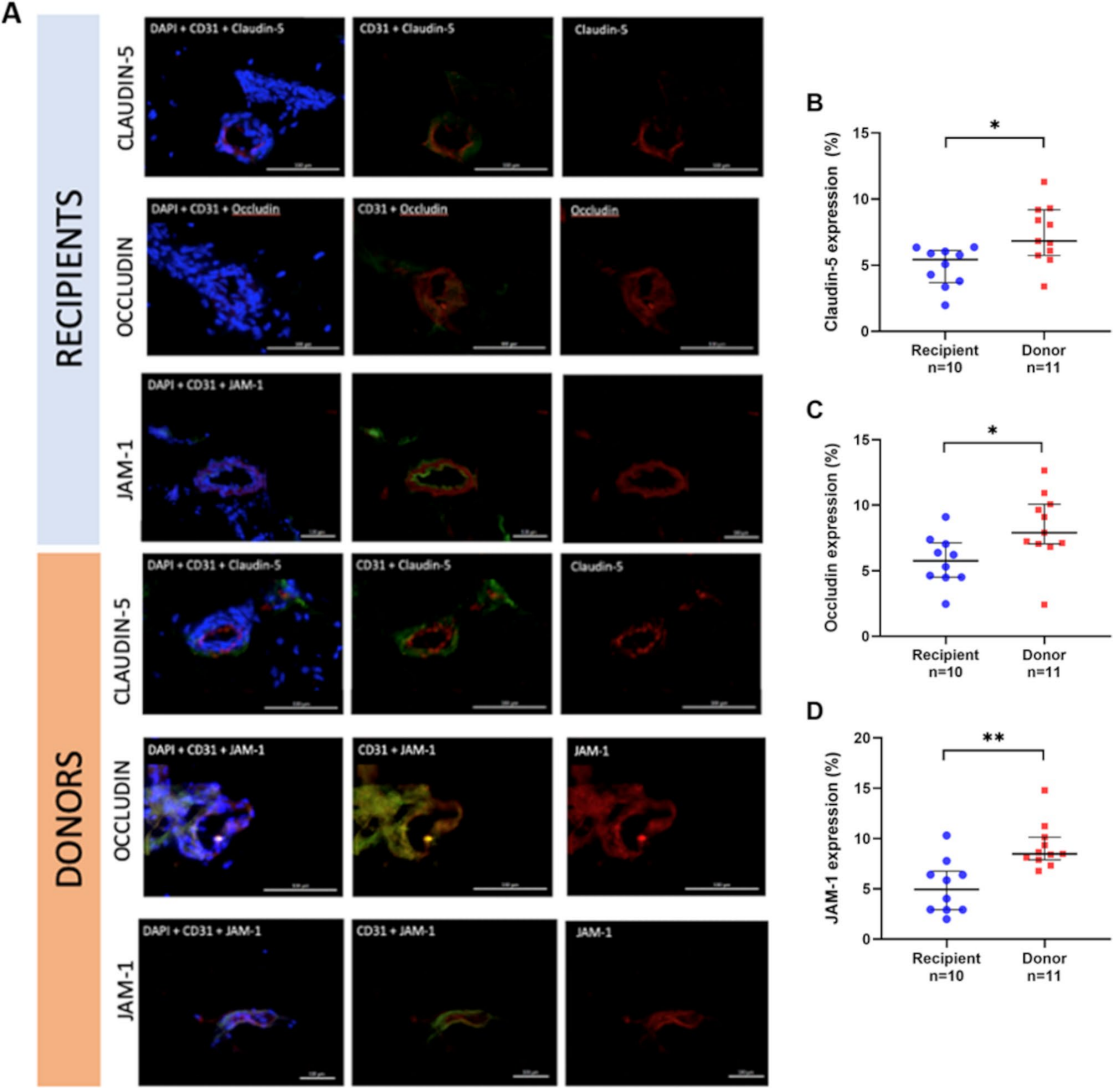
Tight junction protein expression in subcutaneous adipose tissue. (**A**) Immunofluorescence staining of tight junction proteins (red) claudin-5, occludin, and JAM-1, nuclear staining with DAPI (blue), endothelial marker CD31 (green) in subcutaneous tissue of kidney transplant recipients and donors. Bar = 100 µm. Expression of tight junction proteins (B) claudin-5, (**C**) occludin and (**D**) JAM-1 in subcutaneous tissue of kidney transplant recipients and donors. Results expressed in medians and interquartile range. Statistical significance *p < 0.05, **p < 0.01.

The clinical characteristics for kidney transplant and recipient patients who provided subcutaneous fat biopsies are shown in Table 2. The fluorescent tissue staining of the tight junction proteins in microvessels from the subcutaneous tissues of kidney transplant recipients vs donors are shown in Fig. 5A. The expression of tight-junction protein claudin-5 showed lower median expression in kidney transplant recipients compared to donors (5% vs. 7%; p = 0.013) (Fig. 5B). For occludin, kidney transplant recipients also had lower expression compared to donors (6% vs. 8%; p = 0.013) (Fig. 5C). Similarly, JAM-1 kidney transplant recipients also had lower expression compared to donors (5% vs 8%; p = 0.001) (Fig. 5D).

**Table 2:** Clinical characteristics of kidney transplant patients and donors used for immunohistochemistry investigations.

	Recipient	Donor	*p* value
	(n = 10)	(n = 11)	
Age, years	58 (43–67), n = 10	53 (40–63), n = 9	0.59
**Sex, *n* (%)**			
Male	1 (1%)	6 (55%)	
Female	9 (90%)	4 (36%)	
BMI, kg/m^2^	25 (23–29), n = 9	23 (22–28), n = 8	0.49
SBP, mmHg	128 (118–164), n = 9	131 (123–150), n = 8	0.94
DBP, mmHg	77 (70–100), n = 9	82 (73–84), n = 8	0.99
Creatinine, μmol/L	790 (683–1256), n = 9	71 (68–77), n = 7	< 0.001
hsCRP, mg/L	1.8 (0.5–117.5), n = 9	0.4 (0.3–3.9), n = 7	0.30
Albumin, g/L	32 (28–38), n = 9	39 (37–40), n = 8	0.02
Triglyceride, mmol/L	1.3 (1.0–2.1), n = 9	0.8 (0.5–1.0), n = 8	0.007
Cholesterol, mmol/L	5.1 (3.7–5.6), n = 9	4.9 (4.5–6.2), n = 8	0.56
HDL, mmol/L	1.2 (1.0–1.9), n = 9	1.9 (1.4–2.2), n = 8	0.11
HbA1c, %	5.2 (5.0–6.0), n = 9	5.4 (5.3–5.5), n = 6	0.24
**Medications**			
ACEi/ARB, *n* (%)	7 (70)	*Not available*	
Beta-blockers, *n* (%)	5 (5)	*Not available*	
Statins, *n* (%)	4 (4)	*Not available*	

Data are presented as median and quartile range (Q1-Q3). Categorical data are presented as frequency (%). Continuous data analysed by non-parametric Mann–Whitney *U* test.

*BMI* body mass index, *SBP* systolic blood pressure, *DBP* diastolic blood pressure, *hsCRP* high sensitivity C-reactive protein, *HDL* high density lipoprotein, *LDL* low density lipoprotein, *HBA1c* glycated haemoglobin, *IL* interleukin, *ACEi/ARB* angiotensin converting enzyme inhibitor/angiotensin receptor blocker.

**Figure 6:**
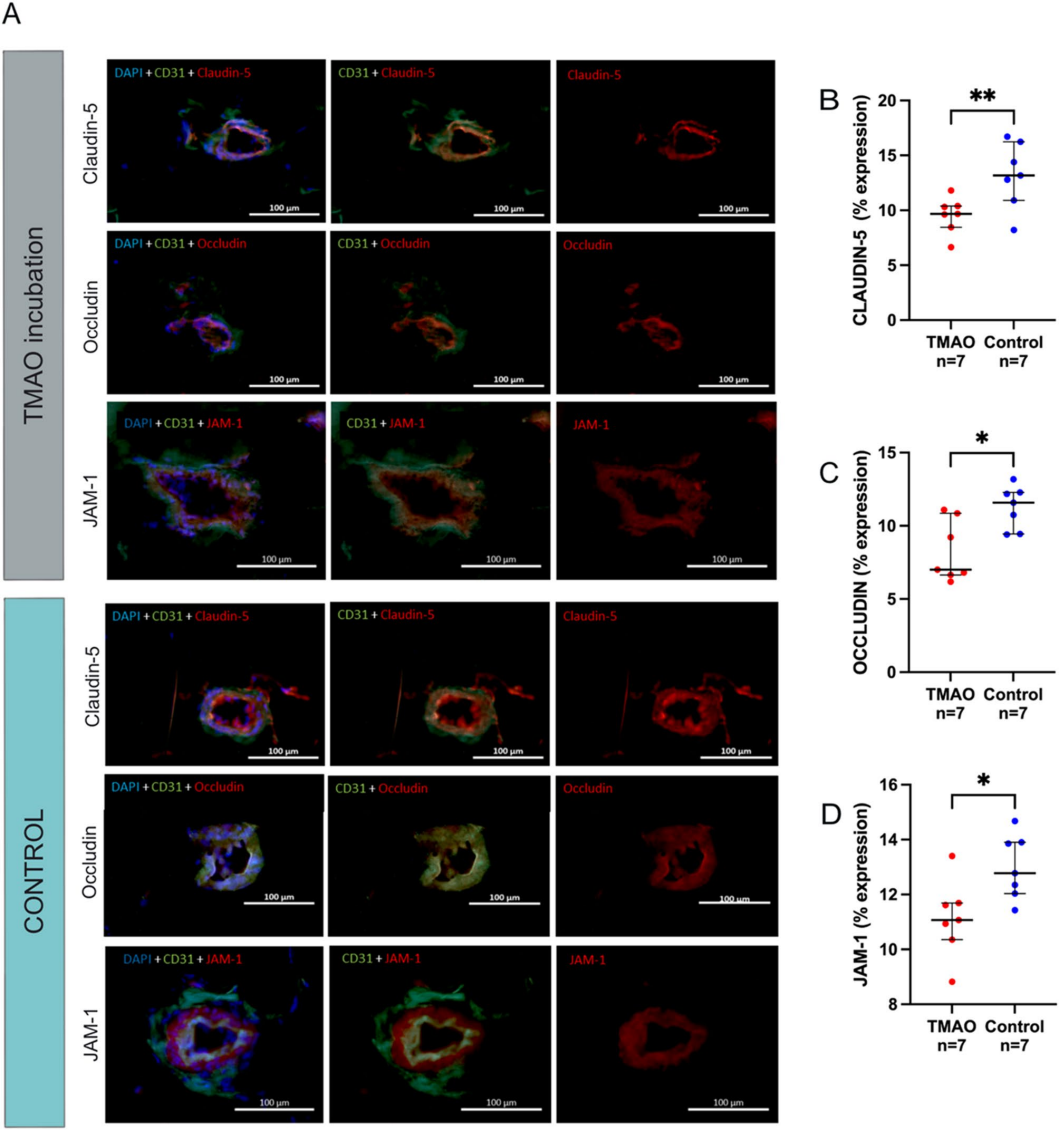
TMAO incubation and tight junction protein expression in subcutaneous adipose tissue. (**A**) Immunofluorescence staining of tight junction proteins (red) claudin-5, occludin, and JAM-1, nuclear staining with DAPI (blue), endothelial marker CD31 (green) in subcutaneous tissue incubated with (**A**) TMAO and (**B**) control media. Bar = 100 µm. Expression of tight junction proteins (**B**) claudin-5, (**C**) occludin and (**D**) JAM-1 in subcutaneous tissue after incubation with TMAO and control media. Results expressed in medians and interquartile range. Statistical significance *p < 0.05, **p < 0.01.

Figure 6A shows the fluorescent tissue staining of tight junction proteins in microvessels from subcutaneous fat tissues from control incubated with TMAO vs control media. The expression of claudin-5 showed lower median expression in TMAO incubated microvessel vs control media (10% vs 13%; p = 0.007) (Fig. 6B). Correspondingly, TMAO incubated microvessels also had lower occludin expression compared to controls (7% vs. 12%; p = 0.034) (Fig. 6C). TMAO incubated microvessel expression of JAM-1 was also lower compared to controls (11% vs 13%; p = 0.011) (Fig. 6D).

## Discussion

Our study supports the suggestion that uraemia may induce disruption in the BBB maintenance as reflected in the altered abundance of brain-specific proteins in the peripheral circulation. In addition, impaired BBB integrity may also be inferred from the decreased expression of tight-junction proteins (claudin-5, occludin, and JAM-1) in the adipose microcirculation—used as a model of the GBB—in KF patients. Decreased expression of tight-junction proteins were also observed in non-CKD adipose microcirculation exposed to the uraemic toxin TMAO, further suggesting that TMAO could act as an important mediator in the disruption of the GBB, and by similarity the BBB. Indeed, TMAO acts not only as a GBB marker but has the potential to impair the BBB maintenance via modulation of endothelial tight junction claudin-5, occludin and JAM-1 in microcirculation that confer BBB integrity.

Our findings imply that BBB damage may occur in uraemic patients due to disruption of the endothelial tight-junction proteins, and the altered levels of circulating protein biomarkers are reflective of the degree of BBB injury^34^. The patients in our study reflect a phenotype that are exposed to a toxic internal environment for years with renal function decline; i.e., increased allostatic load^35^. The exposure and retention of uraemic toxins, alterations in metabolism, inflammation and vascular changes, can lead to the BBB dysfunction, which can reflect persistent neuronal damage that can manifest clinically as depression and cognitive decline^14^. Inflammation being a key feature of the uraemic milieu was confirmed by the increased levels of hsCRP and IL-6 in our prevalent HD patients.

Measurable circulating brain and gut-specific biomarkers that reflect pathological changes in CKD are particularly useful as they could provide a feasible and robust tool to gain insight into the underlying mechanism or pathological processes occurring in uraemia. Our observation of lower BDNF in KF compared to controls is supported by previous work involving KF patients who underwent dialysis^19,36^. In contrast, another study observed increased BDNF levels in HD patients compared to controls, as well as higher BDNF in those diabetic HD patients compared to non-diabetic, postulating that higher BDNF in diabetic HD patients could compensate for insulin resistance and hyperinsulinaemia^37^. The conflicting results of circulating BDNF being lower in our HD cohort compared to controls could be due to the variation in the biological sample used. As serum contain platelets, it may reflect the BDNF levels stored in the platelets^37^ released during the clotting process. In addition, variation of laboratory protocols, handling of samples, fasting state of patient or duration of HD treatment and corresponding time frame of sample collection are also factors to consider. Although no difference in circulating BDNF levels in females and males were observed in our current report, there was a study that noted that female sex was associated with lower plasma BDNF concentration in CKD stage 3–4 patients^38^. Patients in the current study represent latter stages of CKD and have KF, therefore it may be possible that with worsening renal dysfunction the sex difference in levels of BDNF is lost. Hence, a longitudinal study may be necessary to determine the temporal change in BDNF among CKD patients.

Whilst an inverse relationship between BDNF levels with higher depression scores has been reported^19^, possibly reflecting changes in the CNS, our results could not support this. This may be related to the fact that in the present study participants only answered an arbitrary question to report depression, as compared to other studies that have utilised validated clinical scoring system for depression, evaluating of the association between depression and BDNF^19,39^. The implementation of validated depression scoring systems, as well as systems for assessing cognitive function, are advised for future studies assessing BBB integrity in KF patients, unfortunately these cannot be applied retrospectively in our cohort cohorts.

Our result of increased levels of NSE in KF concurs with a previous study where higher serum concentrations of NSE in HD patients were observed^40^, while our results for S100B did not. This suggests that NSE could be valuable in determining possible risks for cerebrovascular events in the uraemic milieu. CKD increases the risk for stroke, and patients with eGFR < 60 ml/min have a greater degree of neurological damage as evidenced by a greater clinical neurological deficit outcome, as analysed with the National Institute of Health Stroke Scale^41^. Stroke risk is particularly high in dialysis patients and people with KF experience a greater stroke mortality^42,43^. When CNS tissue is damaged, thereby disrupting the BBB, there is a pronounced increase in the concentrations of NSE not only in peripheral circulation but also in the cerebrospinal fluid^44^. The support for NSE to be indicative of BBB disruption, also comes from studies including other disease conditions like hypertensive pregnancy complications, as NSE has been studied as a biomarker of CNS injury in pre-eclampsia^45^. Likewise, significant increased levels of NSE seems to be associated with BBB damage and increased the risk of developing cognitive dysfunction^46^. Our finding, that higher levels of NSE in HD patients with poor outcome further strengthens the suggestion that NSE could be a potential marker for assessing the neurologic outcome, though it is important to stress that in acute events (e.g., stroke)^47^ NSE levels could differ from chronic situations like CKD.

Our observation of a 16-fold increase of TMAO levels in KF is supported by previous reports of higher TMAO levels, ranging from 13- to 30-fold, in CKD patients^29,30,48^. This increase is likely related to reduced renal clearance^48^ and/or dietary habits^49^. Whereas we did not observe sex differences in TMAO levels in KF, TMAO levels were higher in non-CKD males vs females, a result reported previously^49^. It is likely that eating habits contribute to the observed differences^49–51^ as sex hormones play a role in appetite regulation^52^, food preferences, and dietary behaviour that may explain sex differences in circulating TMAO. In animal models, the effect of sex on TMAO levels have been related to oestrogen-induced hepatic flavin-containing monooxygenase (FMO) expression being higher in female mice, while testosterone in male mice has been reported to downregulate FMO hepatic activity^53^. However, this may not be a universal suggestion, as in humans no significant difference in FMO activity were found between males and females^54^. Nevertheless, as our women are of menopausal age, it is possible to speculate that the expected reduced oestrogen levels^55–57^ may result in decreased FMO expression, which may be one of factors behind to the lower levels of TMAO in our female controls.. Aside from the role of sex hormones, suggested theories relating to the faster reduction in renal function in males include difference in lifestyle patterns, sex-specific difference in oxidative stress^58–60^, and/or nitric oxide metabolism that are characteristic of endothelial dysfunction in ageing^61^.

Contradictory to other studies showing a positive correlation between TMAO levels and depressive symptoms^55^, our results in KF patients were inconsistent. Our data in KF patients indicate that there might be sex-specific differences between TMAO levels in depressed KF patients, with depressed males having higher TMAO levels compared to females^62,63^. The accumulation of uraemic toxins is believed to weaken the GBB permitting bacterial components and uraemic toxins to enter the systemic circulation through the leaky gut. Since it has been reported that TMAO cross the BBB^56^ it can be speculated that the retention of TMAO in the uraemic milieu may contribute to depression and cognitive dysfunction; common problems in dialysis patients^55,57^. In addition, this gut-derived inflammation will further add to the already existing CKD-associated systemic inflammation, tipping the balance in favour of inflammatory processes, which leads to neuroinflammation whereby proinflammatory cytokines affect the brain signal patterns that cause or maintain the pathology of depression^64^. Paradoxically, our results show a negative correlation between TMAO and inflammation, highlighting the need for further research, however we cannot exclude the significance of the information or the action of uraemic environment on BBB markers.

Human brain tissues and isolation of human brain microvessles are not easily acquired and readily available, therefore limiting research access to human brain endothelial cells. However, studies have shown structural and functional alterations in subcutaneous resistance arteries are predictors of hypertension, ischemic heart disease, heart failure, and cerebral ischemic attacks and renal failure^65,66^. Microvessel remodelling leads to further alterations in renal function and progression of kidney dysfunction and subsequent end-organ damage^65,66^. Therefore, we used resistance size arteries (less than 150 µm internal diameter) in adipose tissue as a surrogate model for the brain microcirculation. Tight-junction proteins, claudin-5, occludin and JAM-1, were expressed at lower levels in uraemic vs control arteries. Our findings supported observations of disruption of tight-junction proteins during conditions like sepsis, where it was reported that intestinal permeability resulted from disruption of the tight-junction structure (claudin 1,3,4,5 and 8) in mice models^67^. A reduced expression of tight-junction proteins claudin-1, occludin and ZO-1 was reported in the colonic mucosa of rat CKD models^68^. Additionally, our result of increased sCD14 levels correlating with TMAO provides further evidence of gut barrier permeability among KF patients. That progression to KF further disturbs the gut flora and further impairs the intestinal barrier allowing for translocation of uraemic toxins into the systemic circulation thus worsening inflammation leading to negative effects and dysfunction in other organs^69,70^. Degradation of occludin in cerebral microvessels in a rat model of ischaemic stroke contributed to BBB disruption and resulted in decreased occludin immunostaining in microvessels, which was reflected by the increased occludin levels in the blood^25^. Similarly, immunostaining and immunoblotting studies of JAM-1 reported a significant decrease in JAM-1 expression in a rat model for cortical cold injury^62^. Furthermore, in human studies of preeclampsia, which is characterised by dysfunctional endothelial cell layer, noted a reduction in claudin 1, 3 and 5 signifying a disrupted endothelial barrier^63^. To further support our theory, we conducted a pilot experiment, using an isolated small artery bioassay to examine the tight-junction protein expression in non-CKD control subcutaneous fat microvessels incubated with TMAO to simulate the exposure of microvasculature to the uraemic environment. In this pilot experiment, we find that in fat microvessels from non CKD controls incubated with TMAO resulted in reduced expression of tight-junction proteins claudin-5, occludin and JAM-1. This reduction in tight junction protein expressions may imply that TMAO can increase endothelial cell layer permeability. This has been recently observed in an in vitro model of the BBB, using human cerebromicrovasular endothelial cell line (hCMEC/D3) monolayers, where TMAO, and its precursor TMA, can alter the permeability of endothelial cell monolayers, with high TMAO concentrations increasing permeability^28^. As CKD—a clinical model of early vascular ageing—is manifested by systemic inflammation^71^, this may pave the way for passing of inflammatory mediators and depression and/or cognitive decline. The observation that TMAO link with cognitive aging in healthy humans support the observation that TMAO cross the BBB^55,57^.

Depression and cognitive decline are closely associated^72^, and CKD patients are prone to developing cognitive decline. Factors contributing to the CKD-associated cognitive decline include uremic toxin accumulation, BP elevation, RAS overactivation, lack of erythropoietin, disturbance in 1,25-dihydroxyvitamin D (1,25(OH)_2_D) as well as microvasculature damage^73^. Cortical areas of the brain in Alzheimer’s disease patients have been linked to dysfunctional tight-junction proteins^74^. The reduced tight-junction proteins leading to loss in the integrity of the BBB allows uraemic toxins to cross the BBB may be a contributing factor in the development of depression and cognitive impairment in CKD.

The results of this study should be interpreted in the light of limitations. Serum samples were taken from HD patients after 1 month of dialysis treatment, this poses a strength in being a unique population, and a weakness in that these patients were on active treatment. In an ideal situation serum samples from baseline prior treatment would have been tested to assess longitudinal alterations, however due to limitations in sample availability this was not possible, In addition, the timing of blood sample collection and patient’s fasting or non-fasting state must be taken into consideration since certain biomarkers like BDNF in particular has been found to be upregulated in fasting state^75^ and has also been observed to have a diurnal variation in men^76^. In the detection of the peripheral biomarker S100B, the assay plate with out-of-range quality control values were included in the results analysis, as excluding it would greatly reduce the number of our sample population. Nevertheless, the observed S100B levels were within similar ranges of previous studies^77–79^. Like many CKD studies, the population is skewed towards males, and although sex disaggregated analyses were performed at all stages, the overall results still may not be generalisable for females. Furthermore, in our outcome for depression, our data is limited by the fact that patients only self-reported presence or absence of depression. Hence a more structured and robust method of depression screening is recommended in future studies. Due to limited sampling, it was not possible to assess for sex differences in the tight-junction protein expression as majority of the biopsies used were dependent on availability of patient specimens from surgical procedures. Further investigations are on-going using a larger cohort to validate our current results in sex specific manner. In addition, the limitation of meeting the ideal criteria for an in-vitro BBB model, due to some morphological or functional differences from other endothelial cells such as having tighter cell junctions and being highly polarised^21^, we cannot assume that the changes in the periphery and BBB are identical, however, the current study provides a platform on which to further investigate tight junctions in CKD. Additional biomarkers in CKD and BBB damage are in also in consideration for future studies. In addition, further investigations are warranted to comprehensively assess the contribution of TMAO to decrease tight junction protein expressions. Such investigations should aim to investigate downstream effectors such PERK, TGFβ/SMAD, and/or NLRP3 inflammasome signalling pathways, which have previously been shown to be affected by TMAO^80–82^. A future prospect also is to conduct a comparative and prospective studies in the assessment of the BBB using similar biomarkers in conjunction with possible brain imaging techniques for assessment of structural abnormalities in relation to cognitive decline among kidney transplant patients. In addition, it would also be interesting to investigate the effects of immunosuppression on the levels of peripheral biomarkers and tight junction proteins in transplant patients as it has been reported in animal studies that glucocorticoids may help to preserve tight junction integrity^83^. Further investigation with regard to BBB damage may also be explored for kidney donor population frequently underappreciated in the studies for long-term outcome.

## Conclusions

This study suggests that the uraemic milieu affects the BBB as reflected by the altered levels of brain specific biomarkers such as NSE and BDNF in the circulation of male and female patients, which may result from the increased leakage of the BBB because of disrupted tight-junction proteins. The GBB is affected by the circulating TMAO which has been shown to induce alteration of proteins claudin-5, occludin, and JAM-1 conferring BBB maintenance. The gut axis is also impaired as reflected by increased TMAO concentrations and is a strong candidate to affect the tight junction protein expression. Further studies are needed to correlate the markers with neurological status as the self-assessed depression was only correlated with high TMAO in males. Since depression screening among KF patients are not routinely performed until symptoms manifest, therefore biomarkers may be a promising tool for possible early screening, however further studies are warranted.

## Materials and methods

### Ethical approval and consent to participate

Informed consent to participate in the study was obtained from all participants. The Regional Ethic Committee of Karolinska Institutet (EPN) approved study protocols, and all studies were carried out in accordance with the Declaration of Helsinki.

### Study population for circulating biomarkers

Circulating biomarker analyses were performed using serum samples from prevalent patients receiving haemodialysis (HD; n = 216), recruited from the Mapping of Inflammatory Markers In Chronic Kidney disease (MIMICK-1) cohort^84^, recruited between October 2003 and September 2004, and non-CKD controls (n = 80) from the PRIMA-control cohort^85^, recruited between February 2003 and May 2013. The control group consisted of an age- and sex-matched population randomly selected from the Stockholm Region, Sweden, by the Statistics Bureau of Sweden—a government agency. No other exclusion criteria other than unwillingness to participate in the study were applied to the selection of control participants. Clinical data were collected from patient medical records at Karolinska University Hospital. Demographic data included, age, BMI, presence of cardiovascular disease and diabetes mellitus and intake of medications such as ACEi/ARB, betablockers and statins. CVD was determined by physician diagnosis from clinical signs of ischemic cardiac diseases, presence of peripheral vascular disease or cerebrovascular disease^86^. CVD and diabetes mellitus status were obtained from patient clinical records.

### Study population for immunohistochemistry

For assessment of tight junctions in microvessels, subcutaneous fat biopsies were obtained from a prospective cohort of living-donor kidney transplant recipients (LD-RTx) (n = 10), and healthy subjects (kidney donors, n = 11) who underwent renal transplantation surgery from May 2019 to June 2020. Additionally, fat biopsies obtained from non-CKD patients (n = 7), who underwent either kidney donation, bariatric surgery or cholecystectomy from November 2020 to December 2020, were used for a TMAO incubation study. All subcutaneous tissue samples were collected on the same day of surgery at Karolinska University Hospital.

### Self-reported depression and mortality outcomes

Assessment of depression among haemodialysis patients was done through a simple patient self-report by responding “yes” or “no” when asked by the nurse coordinator if they were depressed. The medical records were controlled in those reporting depression and if they were taking antidepressant medications. Mortality was recorded during a follow-up period of five years after the conclusion of the three-month study period for HD patients.

### Serum biomarker and biochemical analyses

Serum samples were collected four weeks after the initiation of HD treatment and stored at – 80 °C prior the assessment of circulating biomarkers. Commercially available enzyme-linked immunosorbent assays (ELISA) kits were used to measure the circulating levels of biomarkers in serum samples, including: BDNF (DBD00, R&D Systems, UK), NSE (DENL20, R&D Systems, UK), and S100B (RD192091OOR, Biovendor, Czech Republic). The BDNF and NSE ELISA kits were performed in accordance with manufacturer guidelines. The S100B ELISA kit was performed in accordance with manufacturer guidelines, with the alteration that samples were undiluted. sCD14 levels in HD patients was previously recorded and reported^87^, for measurements in non-CKD controls the same CD14 ELISA kit (DC140, R&D Systems, UK) was used, and run in accordance with manufacturer guidelines. For all kits, patient serum samples were run as singlets to accommodate the sample size. Mass spectrometry was used for the analysis of TMAO in serum samples, and methods were performed as previously described^29^.

Blood samples for biochemical measurements were collected at baseline prior to start of HD. The samples were frozen at – 70 °C if not analysed immediately. Measurements for serum creatinine, high-sensitivity C-reactive protein (hsCRP), interleukin-6 (IL-6), ferritin, albumin, triglyceride, cholesterol, high-density lipoprotein (HDL), and low-density lipoprotein (LDL) were done through routine procedure by the Department of Laboratory Medicine at Karolinska University Hospital as described in previous studies^84,85,88,89^.

### Assessment of tight-junction proteins

Small-vessel disease in the brain is mirrored by small-vessel pathologies in other organs^22^, and resistance arteries are closely associated with blood pressure control, and abnormalities associate with hypertension^65^. Therefore, we used resistance size arteries (less than 150 µm internal diameter) in adipose tissue as a surrogate model for the brain microcirculation^22,65,66^. To examine the explanation(s) behind increased BBB permeability in the uraemic milieu we analysed the expression patterns of tight-junction proteins in microvessels from subcutaneous adipose tissue biopsies from KF patients (kidney transplant recipients) and living controls (kidney donors) using immunohistochemistry. Herein, subcutaneous fat biopsies, measuring approximately 0.5 cm, from LD-RTx recipients (n = 10) and donors (n = 11), were frozen in OCT medium and stored at − 80 °C until sectioning.

In addition, subcutaneous fat biopsies from seven non-CKD patients (kidney transplant donors, bariatric surgery, and cholecystectomy patients; one male and six females, median age 49 (IQR 34–52) years and median BMI 31 (IQR 29–42) kg/m^2^) were used for a TMAO incubational study to assess the effect of TMAO on tight junction proteins. Herein, subcutaneous fat biopsies were incubated with TMAO (100 mmol, 317594-1G, Sigma Aldrich) (DMEM 5% + 100 μM TMAO) or control media (DMEM 5%) for 36 h and frozen in OCT medium and stored at − 80 °C until sectioning. Tissue samples were sectioned at 20 μm thickness and mounted on glass slides that were stored at -20 °C prior to staining.

For fluorescent staining, tissues were fixed with 4% paraformaldehyde for 5 min, followed by washing twice with phosphate-buffered solution (PBS). Antibody blocking for unspecific binding was performed using glycin (100 mM; G58898, Sigma-Aldrich) for 10 min followed by 10% goat serum for one hour at room temperature. Primary antibodies used were claudin-5 (1:200, CSB-PA005507LA01HU, Cusabio Technology, USA), occludin (1:200, CSB-PA016263LA01HU, Cusabio Technology), and JAM-1 (1:400, CSB-PA897579LA01HU, Cusabio Technology), and incubated overnight at 4 °C in 3% Triton and 5% goat serum in 1X PBS. Secondary fluorochrome-conjugated antibody anti-rabbit IgG (1:600, SAB4600407, Sigma-Aldrich) directed against primary antibodies was incubated at room temperature for 45 min. Endothelial marker CD31/PECAM-1 (1:100 553370, BD Biosciences Pharmigen, USA) was double-stained for confirmation of microvascular structures. CD31 antibody was incubated for 1 h followed by secondary fluorochrome-conjugated antibody Alexa Fluor 488 rabbit anti-mouse IgG (1:600, A-11059, Fisher Scientific, Sweden) for 45 min. Washed twice with 3% triton in 1X PBS, counterstaining with DAPI for 5 min, and washed trice with 1X PBS before air-drying. Separate staining for haematoxylin and eosin (H&E) (Mayers HTX 01820, Histolab, Sweden) was done.

Low power (10 × objective) magnification was used to visualise the tissue slides. Microvessels measuring ≤ 150 µm diameter were included for analysis. The images and fluorescent patterns of the antibodies were captured using Carl Zeiss Axio Imager fluorescent microscope. Quantification of the fluorescence and expression of tight junction protein antibodies were analysed with ImageJ software v1.53c.

### Statistical analyses

Statistical analyses were performed using Prism (v8.4, GraphPad, CA, USA), SPSS (v27.0, IBM, NY, USA) and R (v3.6.2) in the RStudio environment (RStudio, MA, USA). The Shapiro–Wilk normality test was performed to assess data distribution, and all subsequent analyses were performed in coherence with data distribution. Non-parametric analysis Mann–Whitney test was used to compare two groups. To test the correlation, Spearman rank correlation test was used. Survival analyses were performed using Cox proportional-hazards regression. Statistical significance was established at p < 0.05.

## Supplementary Information

Supplementary Information.

## Data Availability

The datasets generated and/or analysed during the current study are available from the corresponding author on reasonable request.

## References

[1] Levin A (2017). Global kidney health 2017 and beyond: A roadmap for closing gaps in care, research, and policy. Lancet.

[2] Zoccali C (2017). The systemic nature of CKD. Nat. Rev. Nephrol..

[3] Said S, Hernandez GT (2014). The link between chronic kidney disease and cardiovascular disease. J. Nephropathol..

[4] Jankowski J, Floege J, Fliser D, Böhm M, Marx N (2021). Cardiovascular disease in chronic kidney disease: Pathophysiological insights and therapeutic options. Circulation.

[5] Ebert T (2020). Inflammation and premature ageing in chronic kidney disease. Toxins.

[6] Hobson S, Arefin S, Kublickiene K, Shiels PG, Stenvinkel P (2019). Senescent cells in early vascular ageing and bone disease of chronic kidney disease-a novel target for treatment. Toxins.

[7] Kooman JP, Kotanko P, Schols AMWJ, Shiels PG, Stenvinkel P (2014). Chronic kidney disease and premature ageing. Nat. Rev. Nephrol..

[8] Luksha N (2011). Impaired resistance artery function in patients with end-stage renal disease. Clin. Sci..

[9] Toyoda K (2015). Cerebral small vessel disease and chronic kidney disease. J. Stroke.

[10] Shi Y, Liu Z, Shen Y, Zhu H (2018). A novel perspective linkage between kidney function and alzheimer’s disease. Front. Cell. Neurosci..

[11] Tanaka S, Okusa MD (2020). Crosstalk between the nervous system and the kidney. Kidney Int..

[12] Chen HJ (2018). Re-establishing brain networks in patients with ESRD after successful kidney transplantation. Clin. J. Am. Soc. Nephrol..

[13] Li W, Pan R, Qi Z, Liu K (2018). Current progress in searching for clinically useful biomarkers of blood–brain barrier damage following cerebral ischemia. Brain Circ..

[14] Montagne A (2015). Blood-Brain barrier breakdown in the aging human hippocampus. Neuron.

[15] Basit S, Damholt MB, Wohlfahrt J, Boyd HA (2020). Is kidney disease associated with both Alzheimer’s disease and vascular dementia?. Alzheimer Dement..

[16] Blinov DV, Terentev AA (2013). Characterization of biochemical markers of blood-brain-barrier permeability and the functioning of the central nervous system. Neurochem. J..

[17] Kurajoh M (2017). Plasma brain-derived neurotrophic factor concentration is a predictor of chronic kidney disease in patients with cardiovascular risk factors: Hyogo Sleep Cardio-Autonomic Atherosclerosis study. PLoS ONE.

[18] Lee BH, Kim YK (2010). The roles of BDNF in the pathophysiology of major depression and in antidepressant treatment. Psychiatry Investig..

[19] Kielstein H (2015). Role of the endogenous nitric oxide inhibitor asymmetric dimethylarginine (ADMA) and brain-derived neurotrophic factor (BDNF) in depression and behavioural changes: Clinical and preclinical data in chronic kidney disease. Nephrol. Dial. Transplant..

[20] González-Quevedo A (2016). Serum neuron specific enolase could predict subclinical brain damage and the subsequent occurrence of brain related vascular events during follow up in essential hypertension. J. Neurol. Sci..

[21] Hajal C, le Roi B, Kamm RD, Maoz BM (2021). Biology and models of the blood–brain barrier. Annu. Rev. Bioeng..

[22] Mott M, Pahigiannis K, Koroshetz W (2014). Small blood vessels: Big health problems: National Institute of Neurological disorders and stroke update. Stroke.

[23] O’Rourke MF, Safar ME (2005). Relationship between aortic stiffening and microvascular disease in brain and kidney. Hypertension.

[24] Jia W, Lu R, Martin TA, Jiang WG (2014). The role of claudin-5 in blood-brain barrier (BBB) and brain metastases (review). Mol. Med. Rep..

[25] Pan R (2017). Blood occludin level as a potential biomarker for early blood brain barrier damage following ischemic stroke. Sci. Rep..

[26] van Itallie CM, Anderson JM (2014). Architecture of tight junctions and principles of molecular composition. Semin. Cell Dev. Biol..

[27] Witkowski M, Weeks TL, Hazen SL (2020). Gut microbiota and cardiovascular disease. Circ. Res..

[28] Hoyles L (2021). Regulation of blood-brain barrier integrity by microbiome-associated methylamines and cognition by trimethylamine N-oxide. Microbiome.

[29] Missailidis C (2016). Serum trimethylamine-N-Oxide is strongly related to renal function and predicts outcome in chronic kidney disease. PLoS ONE.

[30] Stubbs JR (2016). Serum trimethylamine-N-oxide is elevated in CKD and correlates with coronary atherosclerosis burden. J. Am. Soc. Nephrol..

[31] Kanitsoraphan C, Rattanawong P, Charoensri S, Senthong V (2018). Trimethylamine N-oxide and risk of cardiovascular disease and mortality. Current Nutrition Reports.

[32] Li D (2018). Trimethylamine-N-oxide promotes brain aging and cognitive impairment in mice. Aging Cell.

[33] Raj DSC (2009). Soluble CD14 levels, interleukin 6, and mortality among prevalent hemodialysis patients. Am. J. Kidney Dis..

[34] Kadry H, Noorani B, Cucullo L (2020). A blood–brain barrier overview on structure, function, impairment, and biomarkers of integrity. Fluids Barriers CNS.

[35] Shiels PG, Stenvinkel P, Kooman JP, McGuinness D (2017). Circulating markers of ageing and allostatic load: A slow train coming. Pract. Lab. Med..

[36] Zoladz JA (2012). Hemodialysis decreases serum brain-derived neurotrophic factor concentration in humans. Neurochem. Res..

[37] Shin SJ, Yoon HE, Chung S, Kim YG, Kim DJ (2012). Plasma brain-derived neurotrophic factor in hemodialysis patients. Int. J. Med. Sci..

[38] Marchelek-Myśliwiec M (2014). Insulin resistance and brain-derived neurotrophic factor levels in chronic kidney disease. Kidney.

[39] Gonul AS (2005). Effect of treatment on serum brain-derived neurotrophic factor levels in depressed patients. Eur. Arch. Psychiatry Clin. Neurosci..

[40] Alpdemir M (2019). Serum neuron specific enolase and S-100B levels in hemodialysis and peritoneal dialysis patients. Eur. Arch. Med. Res..

[41] Lasek-Bal A, Holecki M, Kret B, Hawrot-Kawecka A, Duława J (2014). Evaluation of influence of chronic kidney disease and sodium disturbances on clinical course of acute and sub-acute stage first-ever ischemic stroke. Med. Sci. Monit..

[42] Dad T, Weiner DE (2015). Stroke and chronic kidney disease: Epidemiology, pathogenesis, and management across kidney disease stages. Semin. Nephrol..

[43] de La Mata NL, Masson P, Al-Shahi Salman R, Kelly PJ, Webster AC (2019). Death from stroke in end-stage kidney disease: A population-based study using data linkage. Stroke.

[44] Hajduková L (2015). Biomarkers of brain damage: S100B and NSE concentrations in cerebrospinal fluid: A normative study. BioMed. Res. Int..

[45] Bergman L, Åkerud H (2016). Plasma levels of the cerebral biomarker, neuron-specific enolase, are elevated during pregnancy in women developing preeclampsia. Reprod. Sci..

[46] Nation DA (2019). Blood–brain barrier breakdown is an early biomarker of human cognitive dysfunction. Nat. Med..

[47] Bharosay A (2012). Correlation of brain biomarker neuron specific enolase (NSE) with degree of disability and neurological worsening in cerebrovascular stroke. Indian J. Clin. Biochem..

[48] Pelletier CC (2019). Elevation of trimethylamine-N-oxide in chronic kidney disease: Contribution of decreased glomerular filtration rate. Toxins.

[49] Barrea L (2019). Trimethylamine N-oxide, Mediterranean diet, and nutrition in healthy, normal-weight adults: Also a matter of sex?. Nutrition.

[50] Ebert T (2020). Insights in the regulation of trimetylamine N-oxide production using a comparative biomimetic approach suggest a metabolic switch in hibernating bears. Sci. Rep..

[51] Manippa V, Padulo C, van der Laan LN, Brancucci A (2017). Gender differences in food choice: Effects of superior temporal sulcus stimulation. Front. Hum. Neurosci..

[52] Hirschberg AL (2012). Sex hormones, appetite and eating behaviour in women. Maturitas.

[53] Bennett BJ (2013). Trimethylamine-N-Oxide, a metabolite associated with atherosclerosis, exhibits complex genetic and dietary regulation. Cell Metab..

[54] Xu M (2017). Genetic and nongenetic factors associated with protein abundance of flavin-containing monooxygenase 3 in human liver. J. Pharmacol. Exp. Ther..

[55] Meinitzer S (2020). Sex-specific associations of trimethylamine-N-oxide and zonulin with signs of depression in carbohydrate malabsorbers and nonmalabsorbers. Dis. Mark..

[56] Vernetti L (2017). Functional coupling of human microphysiology systems: Intestine, liver, kidney proximal tubule, blood-brain barrier and skeletal muscle. Sci. Rep..

[57] Brunt VE (2020). The gut microbiome-derived metabolite trimethylamine N-oxide modulates neuroinflammation and cognitive function with aging. GeroScience.

[58] Carrero JJ, Hecking M, Chesnaye NC, Jager KJ (2018). Sex and gender disparities in the epidemiology and outcomes of chronic kidney disease. Nat. Rev. Nephrol..

[59] Swartling O (2021). CKD progression and mortality among men and women: A nationwide study in Sweden. Am. J. Kidney Dis..

[60] Kander MC, Cui Y, Liu Z (2017). Gender difference in oxidative stress: A new look at the mechanisms for cardiovascular diseases. J. Cell Mol. Med..

[61] Herrera MD, Mingorance C, Rodríguez-Rodríguez R, de Sotomayor MA (2010). Endothelial dysfunction and aging: An update. Ageing Res. Rev..

[62] Yeung D, Manias JL, Stewart DJ, Nag S (2008). Decreased junctional adhesion molecule: A expression during blood-brain barrier breakdown. Acta Neuropathol..

[63] Liévano S, Alarcón L, Chávez-Munguía B, González-Mariscal L (2006). Endothelia of term human placentae display diminished expression of tight junction proteins during preeclampsia. Cell Tissue Res..

[64] Jeon SW, Kim YK (2016). Neuroinflammation and cytokine abnormality in major depression: Cause or consequence in that illness?. World J. Psychiatry.

[65] Rizzoni D (2009). Vascular remodeling, macro- and microvessels: Therapeutic implications. Blood Press..

[66] Boari GE (2010). Structural alterations in subcutaneous small resistance arteries predict changes in the renal function of hypertensive patients. J. Hypertens..

[67] Li Q (2009). Disruption of tight junctions during polymicrobial sepsis in vivo. J. Pathol..

[68] Vaziri ND (2012). Disintegration of colonic epithelial tight junction in uremia: A likely cause of CKD-associated inflammation. Nephrol. Dial. Transplant..

[69] Lau WL, Savoj J, Nakata MB, Vaziri ND (2018). Altered microbiome in chronic kidney disease: Systemic effects of gut-derived uremic toxins. Clin. Sci..

[70] Zhou J (2021). Relationship between plasma trimethylamine N-oxide levels and renal dysfunction in patients with hypertension. Kidney Blood Press. Res..

[71] Dai L, Qureshi AR, Witasp A, Lindholm B, Stenvinkel P (2019). Early vascular ageing and cellular senescence in chronic kidney disease. Comput. Struct. Biotechnol. J..

[72] Morimoto SS, Kanellopoulos T, Alexopoulos GS (2014). Cognitive impairment in depressed older adults: Implications for prognosis and treatment. Psychiatr. Ann..

[73] Zhang CY, He FF, Su H, Zhang C, Meng XF (2020). Association between chronic kidney disease and Alzheimer’s disease: An update. Metab. Brain Dis..

[74] Yamazaki Y (2019). Selective loss of cortical endothelial tight junction proteins during Alzheimer’s disease progression. Brain.

[75] Walsh JJ, Edgett BA, Tschakovsky ME, Gurd BJ (2014). Fasting and exercise differentially regulate BDNF mRNA expression in human skeletal muscle. Appl. Physiol. Nutr. Metab..

[76] Piccinni A (2008). Diurnal variation of plasma brain-derived neurotrophic factor (BDNF) in humans: An analysis of sex differences. Chronobiol. Int..

[77] Fan S, Wang H, Yin J (2016). Increase of plasma S100B level in patients with moderate and severe traumatic brain injury. Int. J. Clin. Exp. Pathol..

[78] Kim JK, Kim SG, Kim HJ, Song YR (2012). Serum S100B protein is associated with depressive symptoms in patients with end-stage renal disease. Clin. Biochem..

[79] Bergman L (2014). Plasma levels of S100B in preeclampsia and association with possible central nervous system effects. Am. J. Hypertens..

[80] El-Deeb OS, Atef MM, Hafez YM (2019). The interplay between microbiota-dependent metabolite trimethylamine N-oxide, Transforming growth factor β/SMAD signaling and inflammasome activation in chronic kidney disease patients: A new mechanistic perspective. J. Cell. Biochem..

[81] Chen S (2019). Trimethylamine N-oxide binds and activates PERK to promote metabolic dysfunction. Cell Metab..

[82] Boini KM, Hussain T, Li PL, Koka SS (2017). Trimethylamine-N-oxide instigates NLRP3 inflammasome activation and endothelial dysfunction. Cell. Physiol. Biochem..

[83] Na W (2017). Dexamethasone suppresses JMJD3 gene activation via a putative negative glucocorticoid response element and maintains integrity of tight junctions in brain microvascular endothelial cells. J. Cereb. Blood Flow Metab..

[84] Snaedal S (2016). Dialysis modality and nutritional status are associated with variability of inflammatory markers. Nephrol. Dial. Transplant..

[85] Dai L (2017). Clinical global assessment of nutritional status as predictor of mortality in chronic kidney disease patients. PLoS ONE.

[86] Stenvinkel P, Larsson TE (2013). Chronic kidney disease: A clinical model of premature aging. Am. J. Kidney Dis..

[87] Raj DSC, Shah VO, Rambod M, Kovesdy CP, Kalantar-Zadeh K (2009). Association of soluble endotoxin receptor CD14 and Mortality among Patients Undergoing Hemodialysis. Am. J. Kidney Dis..

[88] Alves FC (2018). The higher mortality associated with low serum albumin is dependent on systemic inflammation in end-stage kidney disease. PLoS ONE.

[89] Sun J (2016). Biomarkers of cardiovascular disease and mortality risk in patients with advanced CKD. Clin. J. Am. Soc. Nephrol..

