# Supplementary material for: Blood–brain barrier and gut barrier dysfunction in chronic kidney disease with a focus on circulating biomarkers and tight junction proteins
Source: Sci Rep. 2022 Mar 15;12:4414. doi: 10.1038/s41598-022-08387-7 (PMC8924178; doi:10.1038/s41598-022-08387-7)
Supplement: Supplementary file 1 — Supplementary Information. [file 41598_2022_8387_MOESM1_ESM.pdf]

ADDITIONAL FILE 1

# Blood-brain barrier and gut barrier dysfunction in chronic kidney disease with a focus on circulating biomarkers and tight junction proteins

Leah Hernandez [1†], Liam J Ward[1†], Samsul Arefin[1], Thomas Ebert[1], Agne Laucyte-Cibulskiene[1,2], GOING-FWD Collaborators*, Olof Heimbürger[1], Peter Barany[1], Lars Wennberg[3], Peter Stenvinkel[1] and Karolina Kublickiene[1]

[1] Department of Clinical Science, Intervention and Technology (CLINTEC), Division of Renal Medicine, Karolinska Institutet, Stockholm, Sweden.

[2] Department of Clinical Sciences, Lund University, Skåne University Hospital, Malmö, Sweden.

[3] Department of Transplantation Surgery, Karolinska University Hospital, Stockholm, Sweden.

* A list of authors and their affiliations appears at the end of main paper.

[†] These authors contributed equally to this work

## List of Additional Tables & Figures

**Table S1:** Sex disaggregated serum concentrations of measured biomarkers in haemodialysis (HD) and non-CKD controls.

**Figure S1:** Analysis of serum S100B biomarker in haemodialysis (HD) and non-CKD controls (control). The number of samples (n) that S100B was detected in is noted. Data presented as median and interquartile range.

**Figure S2:** Self-assessed depression and serum concentration of biomarker in haemodialysis patients; (A) brain-derived neurotrophic factor (BDNF), (B) neuron-specific enolase (NSE), (C) trimethylamine N-oxide (TMAO). The number of samples (n) that each analyte was detected in is noted for each group. Data presented at median and interquartile range.

**Figure S3:** Sex difference in serum concentration of biomarkers in deceased haemodialysis patients; (A) brain-derived neurotrophic factor (BDNF), (B) neuron-specific enolase (NSE), (C) trimethylamine N-oxide (TMAO). The number of samples (n) that each analyte was detected in is noted for each group. Data presented at median and interquartile range.

**Table S1:** Sex disaggregated serum concentrations of measured biomarkers in haemodialysis (HD) and non-CKD controls.

| | Non-CKD control | | *p*-value | HD | | *p*-value |
|---|---|---|---|---|---|---|
| | **Females** | **Males** | | **Females** | **Males** | |
| BDNF, ng/mL | 21.5 (16.4 – 26.8), [n = 23] | 19.4 ( 16.7 – 24.1), [n = 49] | 0.52 | 13.9 (6.5 – 18.9), [n = 89] | 14.1 (9.5 – 19.4), [n = 113] | 0.48 |
| NSE, ng/mL | 3.7 (3.0 – 4.3), [n = 24] | 3.4 (2.9 – 4.4), [n = 56] | 0.75 | 5.3 (4.4 – 6.7), [n = 91] | 5.4 (4.4 – 6.5), [n = 116] | 0.93 |
| S100B, pg/mL | 616.9 (54.9 – 1028.8), [n = 8] | 53.2 (11.1 – 104.5), [n = 13] | 0.13 | 46.2 (23.4 – 240.8), [n = 17] | 24.0 (7.1 – 174.4), [n = 27] | 0.12 |
| TMAO, ng/µL | 0.33 (0.26 – 0.51), [n = 24] | 0.48 (0.35 – 0.64), [n = 56] | **0.036*** | 6.3 (4.1 – 8.6), [n = 66] | 6.7 (4.0 – 12.7), [n = 88] | 0.24 |

Data are presented as median and quartile range (Q1 – Q3).
Statistical comparisons between sexes in each group were performed by non-parametric Mann-Whitney *U* test, *$p < 0.05$.
BDNF – brain-derived neurotrophic factor; CKD – chronic kidney disease; HD – haemodialysis; NSE – neuron-specific enolase; TMAO – trimethylamine N-oxide.

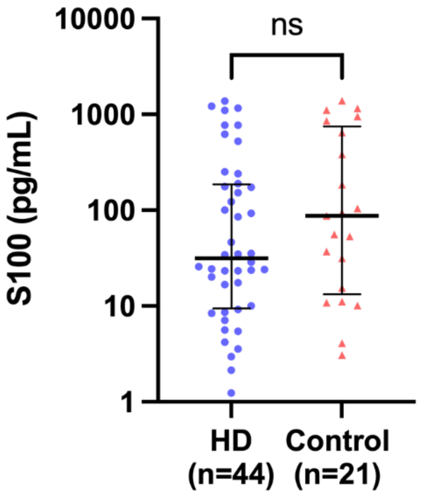


**Figure S1:** Analysis of serum S100B biomarker in haemodialysis (HD) and non-CKD controls (control). The number of samples (n) that S100B was detected in is noted. Data presented as median and interquartile range.

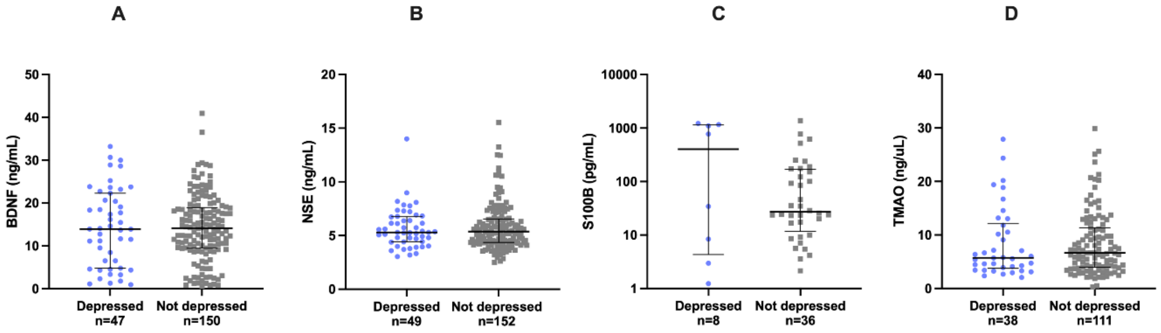


**Figure S2:** Self-assessed depression and serum concentration of biomarker in haemodialysis patients; (A) brain-derived neurotrophic factor (BDNF), (B) neuron-specific enolase (NSE), (D) calcium-binding protein B (S100B) (D) trimethylamine N-oxide (TMAO). The number of samples (n) that each analyte was detected in is noted for each group. Data presented at median and interquartile range.

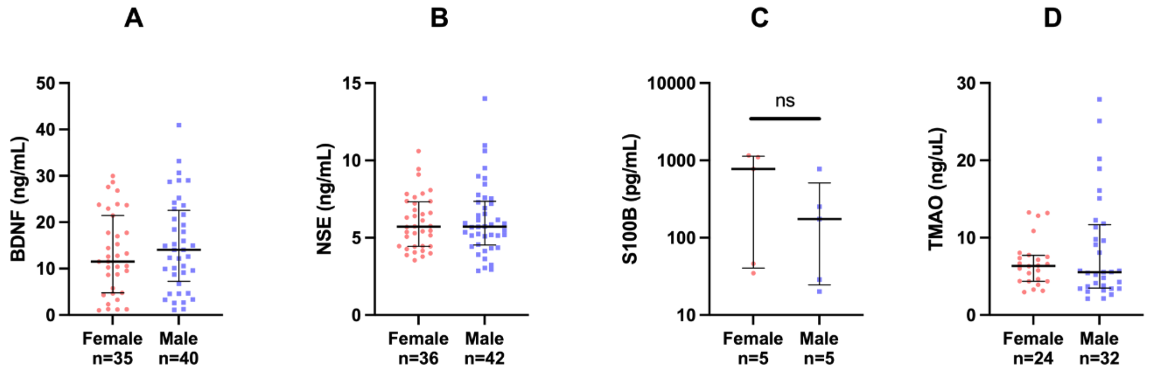


**Figure S3:** Sex difference in serum concentration of biomarkers in deceased haemodialysis patients; (A) brain-derived neurotrophic factor (BDNF), (B) neuron-specific enolase (NSE), (C) calcium-binding protein B (S100B), (D) trimethylamine N-oxide (TMAO). The number of samples (n) that each analyte was detected in is noted for each group. Data presented at median and interquartile range.
